# Genome-wide analysis of *Nicotiana tabacum* IDD genes identifies *NtIDD9* as a regulator of leaf angle

**DOI:** 10.3389/fpls.2024.1496351

**Published:** 2024-12-19

**Authors:** Zefeng Li, Peijian Cao, Huabing Liu, Jianfeng Zhang, Zhaopeng Luo, Hui Zhang, Mingzhu Wu, Xiaodong Xie

**Affiliations:** ^1^ China Tobacco Gene Research Center (CTGRC), Zhengzhou Tobacco Research Institute of China National Tobacco Corporation (CNTC), Zhengzhou, China; ^2^ Second Research Department, Beijing Life Science Academy (BLSA), Beijing, China; ^3^ Technology Center, China Tobacco Zhejiang Industrial, Co Ltd., Hangzhou, China

**Keywords:** IDD family, evolutionary analysis, expression analysis, leaf angle, *Nicotiana tabacum*

## Abstract

The INDETERMINATE DOMAIN (IDD) gene family, encoding a class of C2H2 transcription factor, played diverse roles in land plants. The IDD family in tobacco (*Nicotiana tabacum*) has not been characterized. In this study, 26 NtIDDs were identified in the tobacco genome. Phylogenetic analysis showed that NtIDDs were divided into five groups. Motif analysis revealed that the ID domain was conserved in NtIDDs. Gene duplication analysis demonstrated that segmental/whole-genome duplication and dispersed duplication would have occurred in NtIDDs. *Cis-*element analysis predicted that hormone-, stress-, and development-related elements are located in NtIDD promoters. Expression analysis revealed tissue preference patterns and differential hormone responses in NtIDDs. Further investigations on the function of *NtIDD9* exhibited increased leaf angle degrees in RNA silencing plants. Cellular localization suggested that *NtIDD9* expressed in the endodermis of the leaf petiole base. Subcellular localization analysis revealed that the NtIDD9 protein was located in the nucleus. Hormone quantification found that the levels of auxin, ABA, JA, and GA were significantly changed in *NtIDD9*-silenced plants. Thus, the study suggested that *NtIDD9* played a crucial role in modulation of leaf angle development. Overall, these findings lay foundations for future function and mechanism research on IDDs in tobacco.

## 1 Introduction

Transcription factors (TFs) are proteins involved in the process of gene transcription. TFs function through binding on DNA sequences and activating or repressing the transcription of downstream target genes. TFs are critical components in gene regulatory networks, governing gene expression in various circumstances. A number of TF families have been discovered in plants (Hong, 2016). Cys2His2 (C2H2) zinc finger transcription factors form one of the most significant and expansive families of transcription factors identified to date. The INDETERMINATE DOMAIN (IDD) family, encoding a class of C2H2 transcription factors, constitutes a conserved group across terrestrial plants. IDD genes are characterized by the INDETERMINATE (ID) domain. The ID domain is composed of two C2H2 and two Cys2CysHis (C2CH) zinc finger motifs (ZF1–ZF4) (Colasanti et al., 2006). Prior studies confirmed the involvement of IDDs in transcription regulation, in which C2H2 ZFs are important for DNA binding, whereas C2CH ZFs are necessary for protein–protein interaction (Kozaki et al., 2004; Hirano et al., 2017). In recent studies, with thousands of binding sites captured in the Arabidopsis genome, IDDs were also implied in widespread and complex transcriptional networks (O’Malley et al., 2016).

The IDD family proteins have been shown to participate in diverse processes, including plant development, metabolism, hormone signaling, and environmental stresses (Kumar et al., 2019). The first IDD gene, *ZmIDD1*, was cloned in maize. Analysis indicated that *ZmIDD1* controls the transition to flowering in maize (Singleton, 1946; Colasanti et al., 1998). In rice, *OsID1/Ehd2/RID1* has also been reported to act as a key regulator from vegetative to floral switches (Wu et al., 2008). Gain of function of *OsIDD4* or *OsIDD6* restored flowering of the *rid1* mutant (Deng et al., 2017). *OsIDD10* was found involved in ammonium uptake and nitrogen metabolism in the roots (Xuan et al., 2013a, 2013b). In Arabidopsis, there were 16 IDD genes identified. *AtIDD1* was involved in seed maturation (Feurtado et al., 2011). *AtIDD3* and *AtIDD8* are involved in root development (Ingkasuwan et al., 2012). *AtIDD10* controls root hair cell patterning in the epidermis (Hassan et al., 2010). *AtIDD9* contributes to specifications of epidermal cell fate (Long et al., 2015a, 2015b). *AtIDD8*, *AtIDD14*, and *AtIDD15* play an important role in sugar and starch metabolism (Tanimoto et al., 2008; Ingkasuwan et al., 2012). *AtIDD2*, *AtIDD3*, *AtIDD4*, *AtIDD5*, *AtIDD9*, and *AtIDD10* regulates genes in gibberellin signaling (Fukazawa et al., 2014; Yoshida et al., 2014). *AtIDD14*, *AtIDD15* and *AtIDD16* cooperatively control organ morphogenesis and gravitropic responses by regulating auxin biosynthesis and transport (Cui et al., 2013). *AtIDD14* could respond to cold stress via regulation of *Qua-quine starch* (*QQS*) expression (Li et al., 2009). *AtIDD14* can also interact with *ABFs*/*AREBs*, pivotal genes in the abscisic acid signaling pathway, and positively regulate drought tolerance (Liu et al., 2022). *AtIDD4* acts as a repressor of salt stress in Arabidopsis, and mutations in *AtIDD4* confer enhanced salt tolerance (Rawat et al., 2023).

Leaf angle refers to the inclination formed between the leaf midvein and the stem, which is an important trait of plant architecture. The leaf angle has direct impacts on plant density, photosynthetic light use efficiency, stress tolerance, and, consequently, the overall yield of the plant (Cao et al., 2022). Until date, numerous genes have been reported to regulate leaf angle in plants. Among them, IDD genes are recognized to exert essential roles in leaf angle regulation. For example, in Arabidopsis, *SHOOT GRAVITROPISM5* (*SGR5*), also named as *AtIDD15*, was initially detected able to change the shoot growth orientation by altering gravity sensing (Morita et al., 2006). Further characterization on its close homologs revealed that *AtIDD14*, *AtIDD15*, and *AtIDD16* cooperatively regulate the orientation angles of both branches and siliques (Cui et al., 2013). In rice, the homologue of *AtIDD15*, *OsIDD14*/*Loose Plant Architecture1* (*LPA1*) modulates rice tiller and leaf angle by controlling the adaxial growth at the tiller node and lamina joint (Wu et al., 2013).

Tobacco (*Nicotiana tabacum*) is an important economic crop and cultivated widely. Growth and development, metabolism, and stress resistance are all key aspects for tobacco plant production. As versatile functions disclosed in model plants, the IDD family is deemed as a potential target for crop improvement (Coelho et al., 2018). At present, genome-wide identification and analysis of IDD family have been carried out in several plant species, such as rice, maize, cotton, rapeseed, pear, and apple (Fan et al., 2017; Ali et al., 2019; Su et al., 2019; Zhang et al., 2020; Sun et al., 2022; Feng et al., 2023). However, the IDD family in tobacco has not been characterized and reports on their functions are quite rare. Tobacco is also known as a leaf-harvesting crop. Adjusting the size of the leaf angle is a crucial strategy for managing both the yield and quality of tobacco leaf production. Nevertheless, studies on the genetic basis of leaf angle regulation in tobacco are limited. Although leaf angle regulatory roles of IDDs were characterized in Arabidopsis and rice, they were not fully investigated in tobacco yet.

In the current study, IDD members were identified in tobacco genome; their phylogeny, gene structures, protein motifs, chromosome distributions, duplications, and promoter *cis*-elements were analyzed. IDD gene expression profiles in different tissues and hormone treatments were also investigated. In addition, the role of *NtIDD9* in leaf angle regulation was further explored. These results provide extensive understanding of the IDD family in tobacco and will facilitate the investigation of functions and regulatory mechanisms associated with IDD members.

## 2 Materials and methods

### 2.1 Identification of NtIDD genes

AtIDD protein sequences were used as queries to search homologous genes in tobacco genome (Edwards et al., 2017) with BLASTP (E value<100) (Camacho et al., 2009). InterPro (v93, https://www.ebi.ac.uk/interpro/) (Paysan-Lafosse et al., 2023) was used to predict the ID domain (IPR031140). Sequences were subjected to manual curation. Genes with incomplete ID domains were removed. The molecular weights and isoelectric points were calculated with EMBOSS (Rice et al., 2000).

### 2.2 Construction of phylogenetic tree

IDD protein sequences in Arabidopsis thaliana, rice, and maize were collected from Phytozome (v10, https://phytozome-next.jgi.doe.gov/) (Goodstein et al., 2012). Multiple-sequence alignments were carried out by MAFFT (v7.520) (Katoh and Standley, 2013). A phylogenetic tree was constructed by MEGA (v7.0.21) (Kumar et al., 2016) using the maximum likelihood (ML) method with bootstrap value 1,000. The tree was edited on iTOL (https://itol.embl.de/) (Letunic and Bork, 2021).

### 2.3 Gene structure and synteny analysis

Gene structure annotations were obtained from the GFF3 file of tobacco genome annotation. MEME (v4.9.1) (Bailey et al., 2015) was used to discover motifs in NtIDD protein sequences with parameters (minw = 8, maxw = 50, nmotifs = 10). The gene structure and motifs were plotted with the custom Python script.

The genomic locations of NtIDDs were retrieved from the tobacco genome GFF3 file (Edwards et al., 2017 version, http://solgenomics.net/ftp/genomes/Nicotiana_tabacum/edwards_et_al_2017). Synteny analysis was performed using MCScanX (Wang et al., 2012); minimum five genes were required to call a syntenic block. The types of duplication were identified using the duplicate_gene_classifier program resided in the MCScanX package. Genome distributions and syntenic blocks were visualized using Circos software (Krzywinski et al., 2009).

### 2.4 *Cis*-element prediction

For *cis*-element prediction, 1.5-kb upstream sequences of NtIDD genes were extracted and submitted to PlantCARE (https://bioinformatics.psb.ugent.be/webtools/plantcare/html/) (Lescot et al., 2002). *NtIDD1* was not performed, due to too many ambiguous bases (N) in its promoter region. After prediction, filtering was further carried out, and elements involved in three categories (hormone responsive, stress responsive, and development related) were considered for analysis.

### 2.5 RNA-Seq data analysis

Public RNA-Seq data generated by a previous study (GenBank accession code: SRP029183) (Sierro et al., 2014) were used for tissue expression analysis. The data were mapped to tobacco genome with HISAT2 (v2.1.0) (Kim et al., 2019). Gene expression levels (FPKM, Fragments Per Kilobase of transcript per Million mapped reads) were estimated using StringTie2 (v2.1.7) (Pertea et al., 2015).

### 2.6 Plant materials and growth conditions

Tobacco plants intended for hormone treatments were cultivated in a greenhouse maintained at temperatures of 28/24°C, with regulated light conditions of 16 h of light and 8 h of darkness. 3-week-old tobacco seedlings were soaked into liquid medium containing 50 μM methyl jasmonate (MeJA), 10 μM abscisic acid (ABA), 10 μM salicylic acid (SA), 10 μM gibberellin acid (GA), 10 μM 6-benzylaminopurine (6-BA, cytokinin, CK), and 5 μM GR24 (strigolactone, SL), where they were cultured for a duration of 5 h. Control seedlings were treated with a 1% (v/v) dimethyl sulfoxide (DMSO) solution.

### 2.7 Quantitative real-time PCR

Samples were collected with three biological replicates. Total RNA of different samples was extracted with RNA Kit (Imagene, Beijing, China) according to the instruction. The DNA was firstly removed using RNase-free DNase I (Takara, Beijing, China). Then, high-quality RNA was used for cDNA synthesis using Reverse Transcriptase M-MLV (Takara). The quantitative real-time PCR (qRT-PCR) was quantified on a LightCycler^®^ 96 Real-Time PCR System. The reaction program was set as the following: 95°C for 30 s, 40 cycles of 95°C for 10 s, 60°C for 30 s. The *NtGAPDH* gene was used as reference gene to standardize the expression level with the 2^−△△CT^ method. The primer sequences are listed in **Supplementary Table 6**.

### 2.8 Transgenic plant construction

To construct RNA interference (RNAi) plants, the full-length coding sequence (CDS) of the *NtIDD9* gene was firstly amplified. Subsequently, the derived PCR fragment was connected to the pBWA(V)HS vector through homologous recombination. The constructs were then transformed into tobacco with the help of Agrobacterium tumefaciens strain GV3101.

### 2.9 Cellular and subcellular localization

For cellular localization, RNA *in situ* hybridization was conducted according to the manufacturer’s protocol (Servicebio, Wuhan, China). The leaf petiole base tissue was fixed with *in situ* hybridization fixative (plant) and embedded in wax. Paraffin blocks were sliced 6 μm thick using a slicing machine. The slides were then dewaxed, dehydrated, digested, and hybridized to probes. After washing and dropping anti-Digoxin antibody, BCIP/NBT solution was used for chromogenic staining. Under microscopic observation, blue and blue-purple were interpreted as positive hybridization.

To verify the subcellular location of NtIDD9 protein, the full-length coding sequence (CDS) without stop codon was cloned into the pC1300 and C-terminal fused with enhanced green fluorescent protein (GFP). The product was reclaimed from the recombination ligation gel, and the recombinant plasmid obtained from the ligation was introduced into *Agrobacterium tumefaciens* strain GV3101. The vector harboring 35S::GFP-NtIDD9 and control vector were infiltrated into *Nicotiana benthamiana* leaves. After 24  h, fluorescence images were observed using a confocal microscope (FV1200 Olympus, Japan).

### 2.10 Plant hormone quantification and analysis

The samples were frozen in liquid nitrogen and crushed into fine powder. 50 mg of powdered samples was used to extract endogenous hormones with 1 mL of methyl−tert−butyl−ether (MTBE) solution containing MTBE, methanol, and water in a ratio of 15:4:1. After centrifugation, supernatant was collected to perform liquid chromatography with tandem mass spectrometry (LC-MS/MS) analysis. The data were captured on instrument system UPLC (ExionLC™ AD) coupled with MS/MS (QTRAP^®^ 6500+). By using each standard hormone calibration curve, the levels of seven endogenous hormones including auxins, cytokinins (CKs), gibberellin acids (GAs), methyl jasmonates (MeJAs), salicylic acids (SAs), abscisic acids (ABAs), and ethylenes (ETHs) were quantified. To get statistically meaningful results, three biological replicates were carried out. The differential analysis were determined using *t*-test with false discovery rate (FDR) < 0.05 and |log2(fold change)| ≥1.

## 3 Results

### 3.1 Identification and phylogeny of IDD genes in tobacco

**Figure 1:**
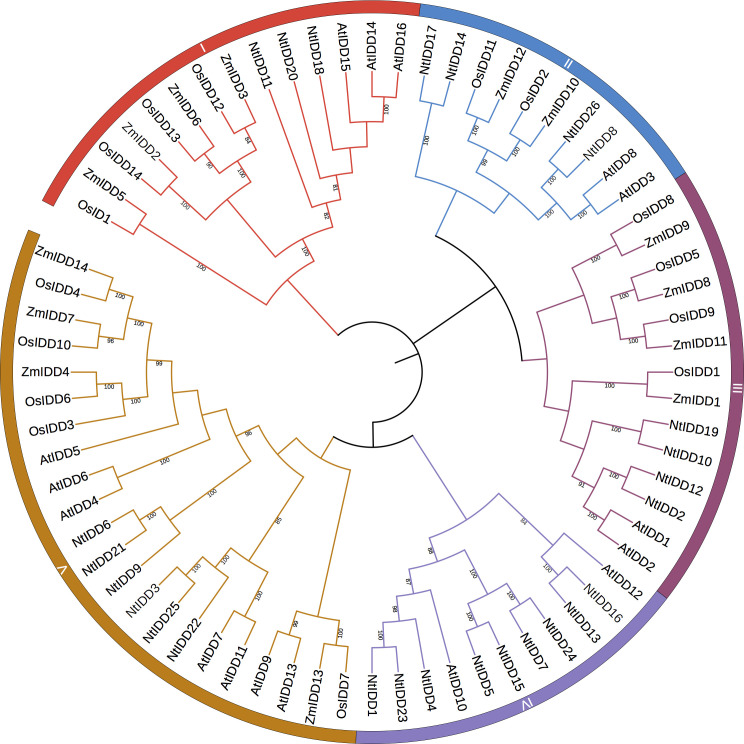
Phylogenetic tree of NtIDDs. Nt, *Nicotiana tabacum*; At, *Arabidopsis thaliana*; Zm, *Zea mays*; Os, *Oryza sativa*.

Based on homology searching and ID domain prediction, 36 potential tobacco IDD genes were firstly screened (**Supplementary Table 1**). After removing genes without complete ID domains, totally 26 IDDs were identified in the tobacco genome. The characteristics of NtIDD genes are listed in **Table 1** and **Supplementary Table 2**. In brief, the peptide lengths were ranged from 382 to 542. The molecular weights (Mw) were distributed from 44 kDa to 59.5 kDa. The isoelectric points (pI) were between 8.5 and 10.1. NtIDD protein sequences, together with IDDs from *Arabidopsis thaliana*, rice, and maize, were used to construct phylogenetic tree (**Figure 1**). Based on the tree, NtIDDs could be classified into five groups: group I (*NtIDD11/20/18*), group II (*NtIDD12/2/19/10*), group III (*NtIDD17/14/26/8*), group IV (*NtIDD24/7/15/5/4/23/1/16/13*), and group V (*NtIDD25/3/22/21/6/9*).

**Table 1:** Summary characteristics of NtIDDs.

Name	ID	Peptide length	Mw(kDa)	pI
NtIDD1	Nitab4.5_0000669g0050	432	47.9	9.6
NtIDD2	Nitab4.5_0002128g0060	536	55.8	8.6
NtIDD3	Nitab4.5_0001295g0240	538	58.5	8.8
NtIDD4	Nitab4.5_0001192g0030	491	54.1	9.5
NtIDD5	Nitab4.5_0000196g0040	522	57.4	9.5
NtIDD6	Nitab4.5_0000944g0030	509	55.5	9.1
NtIDD7	Nitab4.5_0000650g0060	472	52.2	9.5
NtIDD8	Nitab4.5_0005191g0020	444	49.4	9.0
NtIDD9	Nitab4.5_0000129g0500	408	45.5	10.1
NtIDD10	Nitab4.5_0000110g0440	521	56.2	9.0
NtIDD11	Nitab4.5_0001735g0010	382	44.0	8.5
NtIDD12	Nitab4.5_0001279g0070	537	56.0	8.8
NtIDD13	Nitab4.5_0000459g0080	506	56.0	8.5
NtIDD14	Nitab4.5_0001697g0070	520	57.1	9.5
NtIDD15	Nitab4.5_0000106g0080	540	59.5	9.6
NtIDD16	Nitab4.5_0004115g0010	501	55.7	8.5
NtIDD17	Nitab4.5_0001447g0010	505	55.5	8.7
NtIDD18	Nitab4.5_0000521g0010	397	44.5	8.8
NtIDD19	Nitab4.5_0003246g0020	522	56.3	9.0
NtIDD20	Nitab4.5_0004454g0060	443	49.8	8.9
NtIDD21	Nitab4.5_0004543g0030	515	55.9	8.9
NtIDD22	Nitab4.5_0007697g0010	538	59.2	8.9
NtIDD23	Nitab4.5_0008025g0010	493	54.3	9.6
NtIDD24	Nitab4.5_0010509g0010	469	51.9	10.0
NtIDD25	Nitab4.5_0014751g0010	542	58.9	8.6
NtIDD26	Nitab4.5_0022126g0010	453	50.4	8.9

### 3.2 Gene structure and motif distribution in NtIDDs

**Figure 2:**
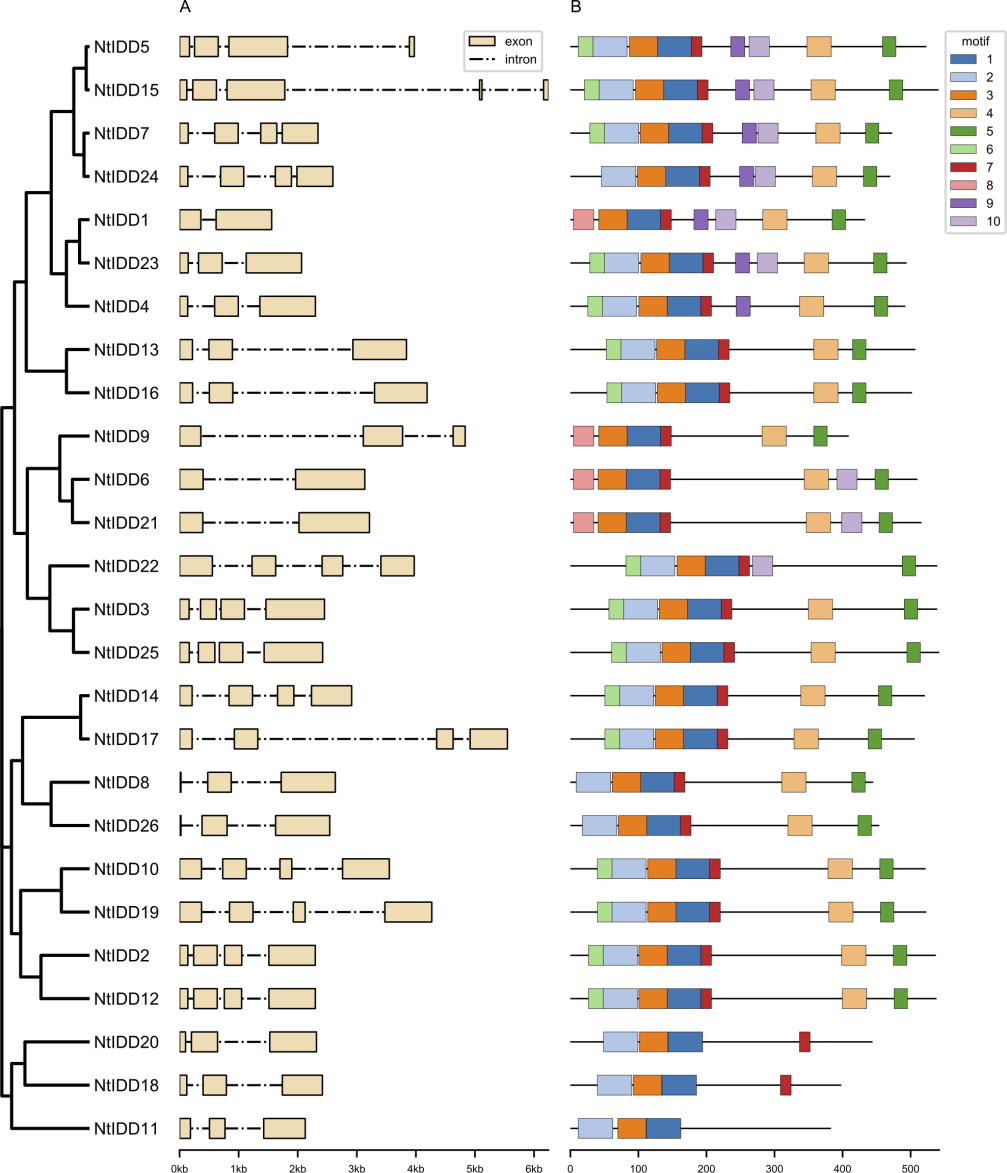
NtIDD gene architectures **(A)** and motif distributions **(B)**.

**Figure 3:**
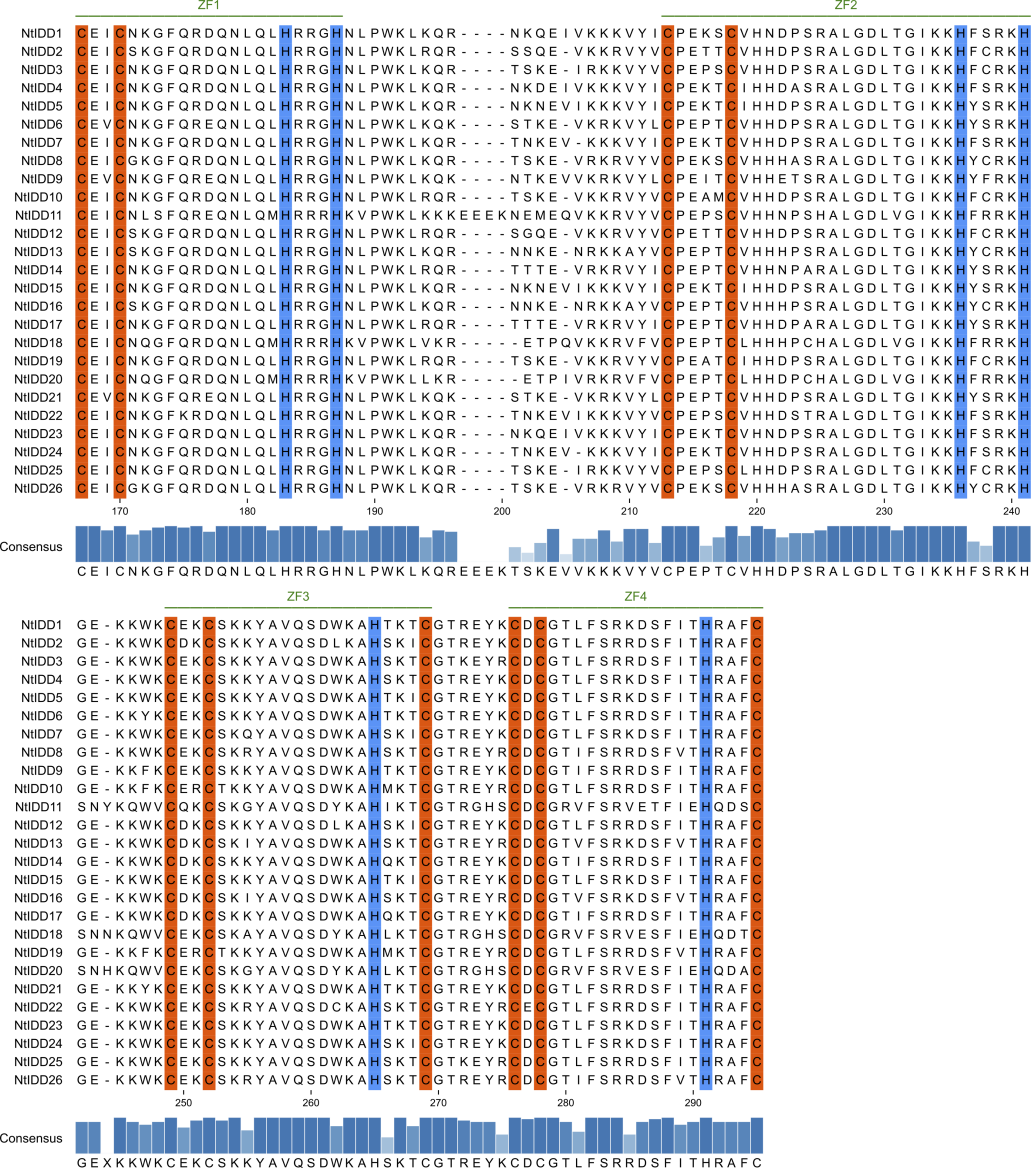
Multiple-sequence alignment of NtIDD proteins. Zinc finger motifs (ZF1, ZF2, ZF3, ZF4) are marked with green lines. The conserved amino acids cysteine and histidine are highlighted with orange and blue colors, respectively.

Most of NtIDD genes comprised three to four exons (**Figure 2A**). *NtIDD15* contains five exons, whereas *NtIDD1*, *NtIDD6*, and *NtIDD21* possess only two exons. The intron length of NtIDD genes varied greatly. The shortest and longest introns were found in *NtIDD25* and *NtIDD15*, respectively. Using MEME, 10 motifs were identified in NtIDD protein sequences (**Figure 2B**). Among them, motif 1/3 were found in all NtIDDs, motif 2/4/5/6/7 were distributed in most of NtIDDs, motif 8 was specifically found in *NtIDD1/9/6/21*, motif 9 was found in *NtIDD5/15/7/24/1/23/4*, and motif 10 was found in *NtIDD5/15/7/24/1/23/6/21/22*. Motifs 1/2/3/8 were associated with the ID domain, which consists of two C2H2 zinc fingers and two C2CH zinc fingers (**Figure 3**).

### 3.3 Location and duplication of NtIDDs

**Figure 4:**
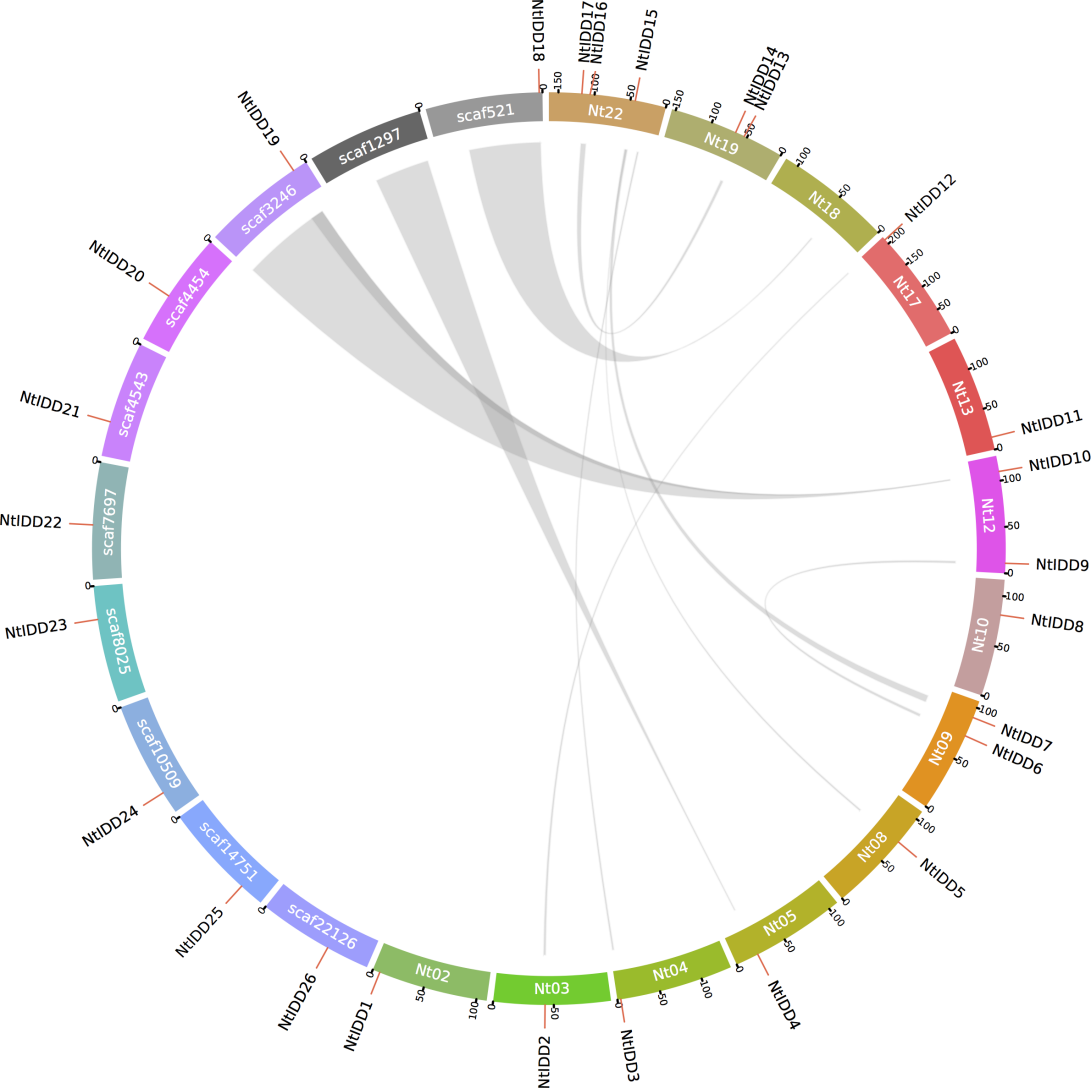
Genome distribution and synteny among NtIDDs. Nt, chromosome; scaf, scaffold. Gray links represent syntenic blocks.

By investigating distributions of NtIDDs in the genome, 17 genes were positioned on chromosomes, and 9 genes were located on scaffolds (**Figure 4**). Using MCScanX, syntenic blocks were identified and the types of duplication were classified. There were 14 NtIDDs that were found involved in syntenic blocks (**Figure 4**) and therefore were deemed as segmental and/or whole-genome duplications (WGD). There were six syntenic gene pairs detected among these NtIDDs (*NtIDD2/12*, *NtIDD5/15*, *NtIDD6/9*, *NtIDD7/15*, *NtIDD10/19*, *NtIDD13/16*). The other 12 NtIDDs distributed out of synteny, and they were categorized as dispersed duplications.

### 3.4 *Cis*-elements in the upstream of NtIDDs

**Figure 5:**
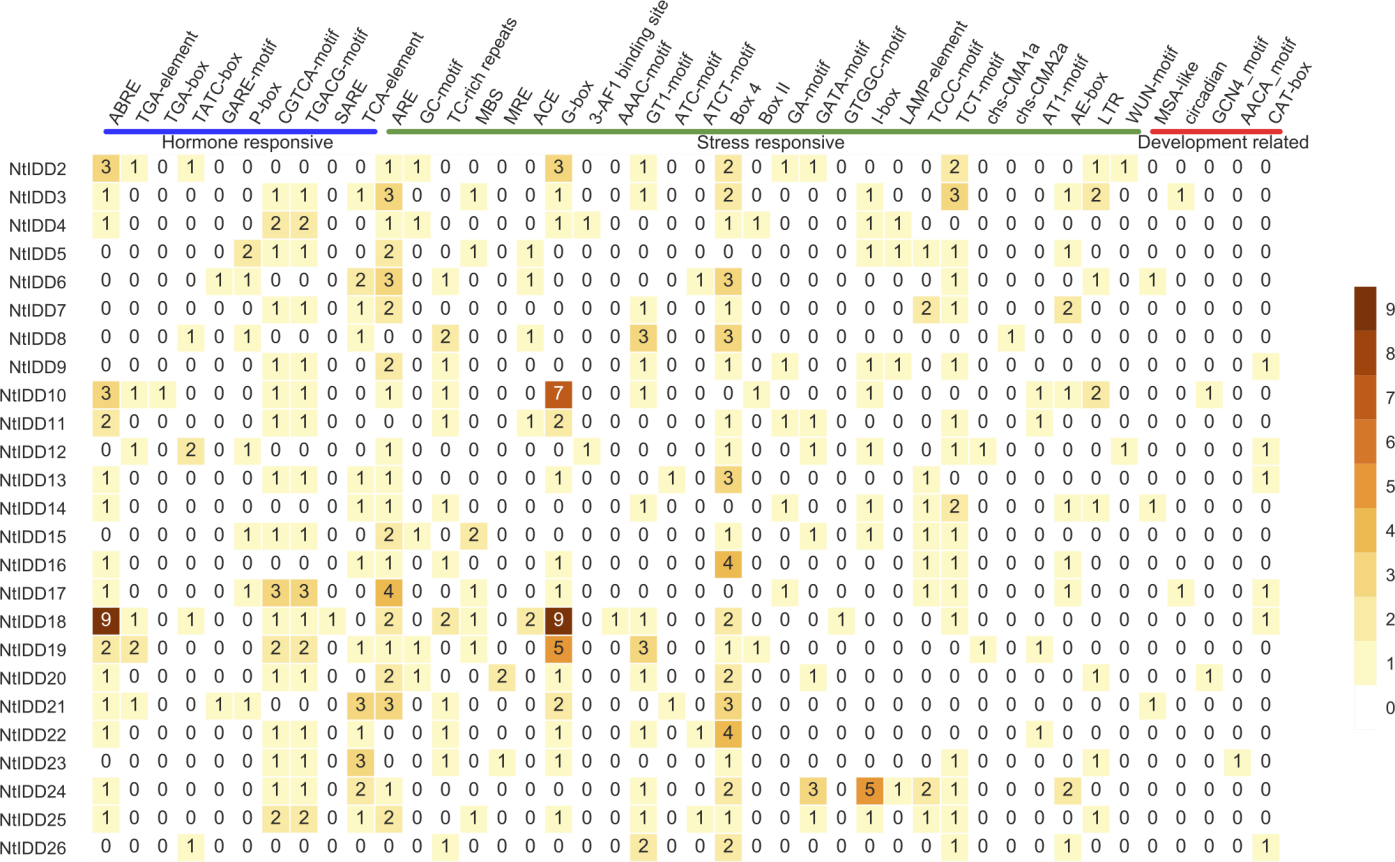
Number of *cis*-elements in NtIDD promoters.

In order to gain more insights on regulatory mechanism, the 1.5-kb upstream sequences of NtIDDs were extracted, and their *cis*-elements were predicted. There were total 42 elements identified (**Figure 5**; **Supplementary Table 3**). Among them, 10 elements were hormone responsive, 27 elements were stress responsive, and 5 elements were development related.

In the hormone-responsive elements, ABRE (abscisic acid responsive), CGTCA-motif/TGACG-motif (MeJA responsive), and TCA-element (salicylic acid responsive) were detected in most of NtIDDs. TGA-element/TGA-box (auxin responsive), TATC-box/GARE-motif/P-box (gibberellin responsive), and SARE (salicylic acid responsive) were discovered in few NtIDDs, such as *NtIDD2* and *NtIDD10*.

In the stress-responsive elements, ARE (anaerobic induction), TC-rich repeats (defense and stress responsive), and G-box/GT1-motif/Box 4/TCT motif (light responsive) were identified in most of NtIDDs. GC-motif (anoxic induction), MBS (drought inducibility), LTR (low-temperature responsive), WUN-motif (wound responsive), and the remaining elements (light responsive) were distributed in several NtIDDs, like *NtIDD2* and *NtIDD4*.

For the development-related elements, MSA-like (cell cycle regulation) was identified in *NtIDD6/14/21*, circadian (circadian control) was found in *NtIDD3* and *NtIDD17*, GCN4_motif/AACA_motif (endosperm expression) were detected in *NtIDD10/20/23*, and CAT-box (meristem expression) was discovered in *NtIDD9/12/13/17/18/26*.

### 3.5 Tissue expression patterns of NtIDDs

**Figure 6:**
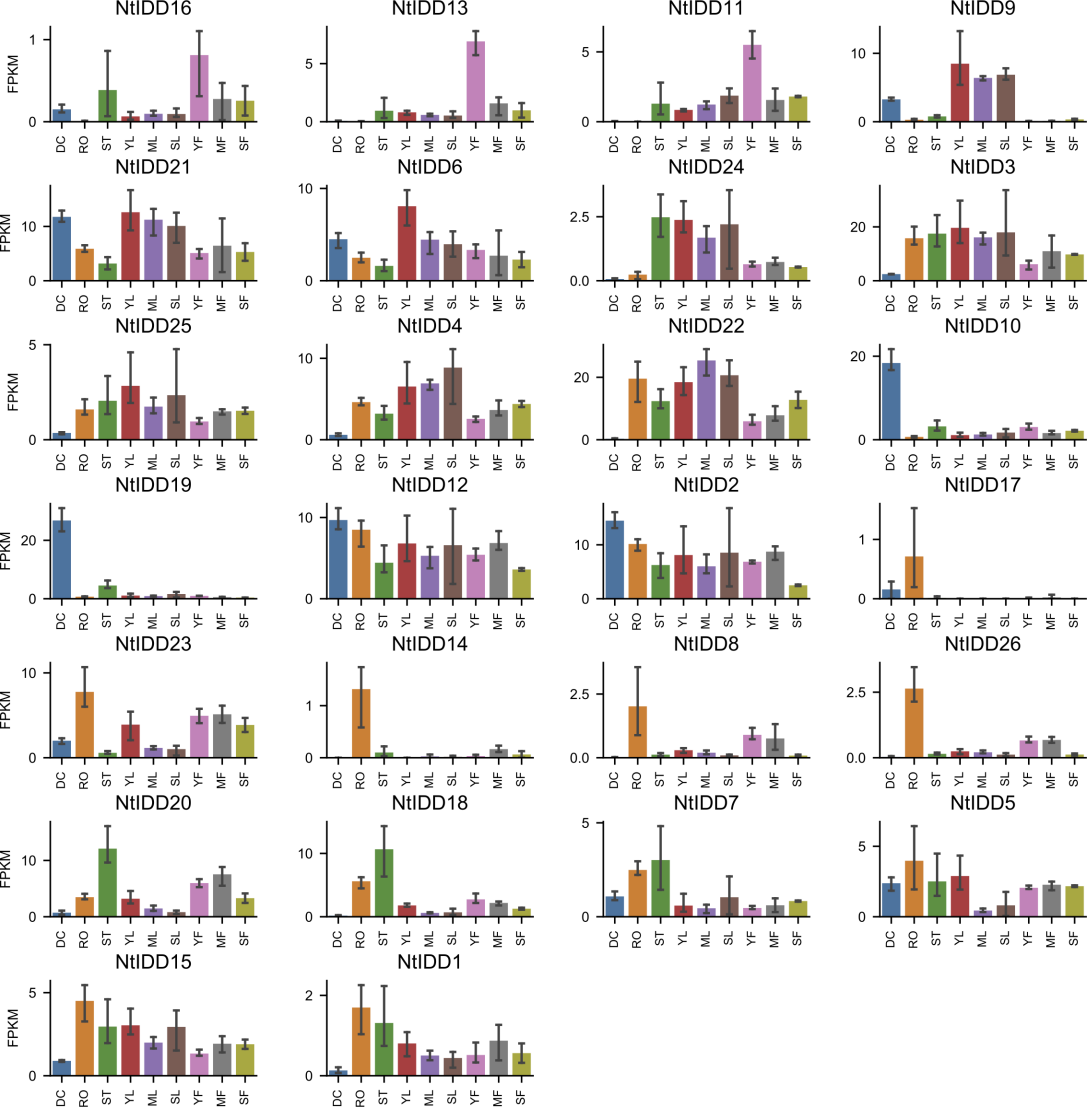
Tissue expression levels of NtIDDs. DC, dry capsule; RO, root; ST, stem; YL, young leaf; ML, mature leaf; SL, senescent leaf; YF, young flower; MF, mature flower; SF, senescent flower. The error bar represents the mean ± SE.

**Figure 7:**
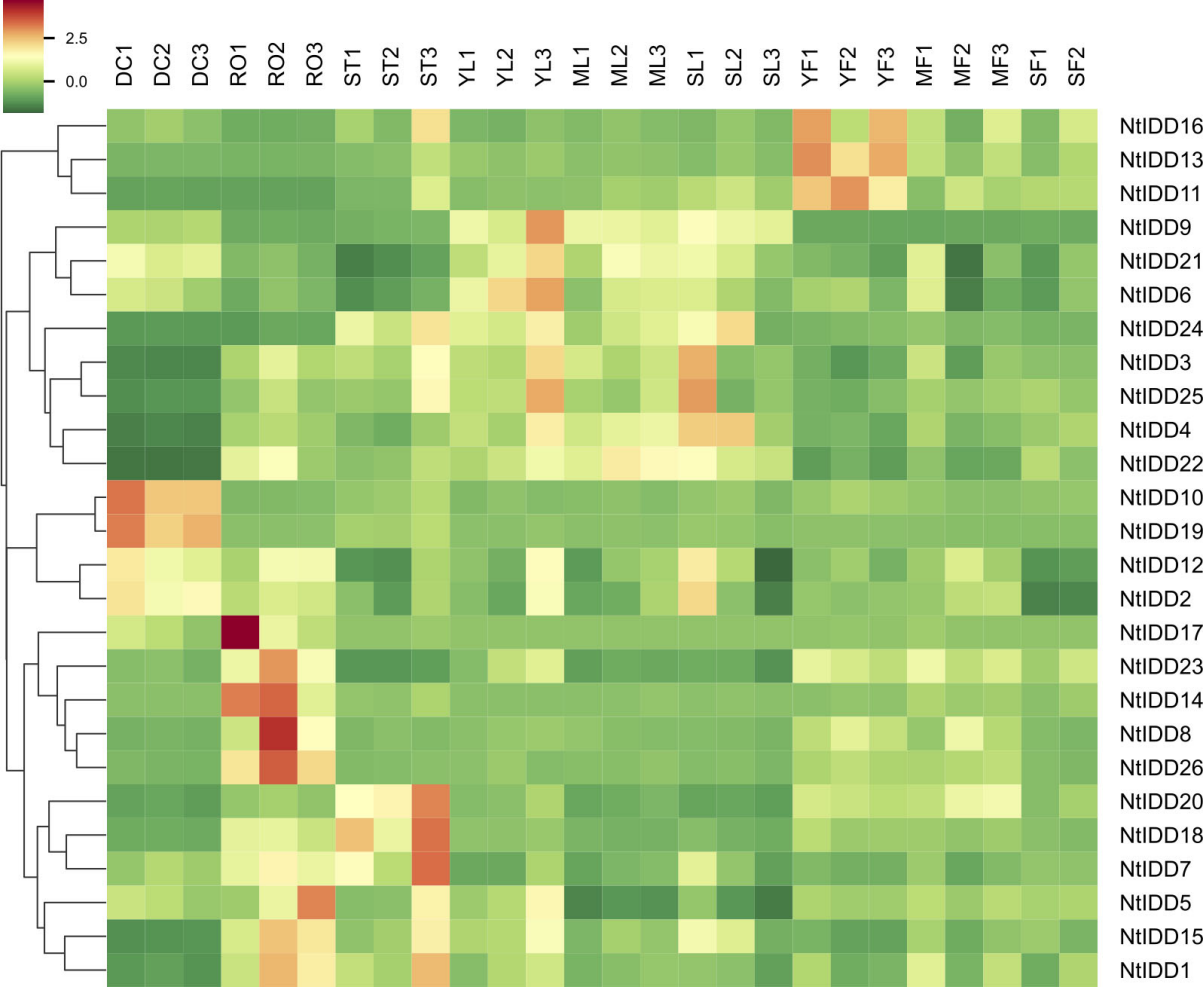
Heatmap of NtIDD tissue expression clustering. DC, dry capsule; RO, root; ST, stem; YL, young leaf; ML, mature leaf; SL, senescent leaf; YF, young flower; MF, mature flower; SF, senescent flower. The numbers (1–3) represent biological replicates.

Utilizing public RNA-Seq data, their expressions in nine tissues (Dry Capsule, Root, Stem, Young Leaf, Mature Leaf, Senescent Leaf, Immature Flower, Mature Flower, Senescent Flower) were investigated. In general, NtIDDs expressed throughout all the nine tissues (**Figure 6**; **Supplementary Table 4**). Most of NtIDDs expressed in multiple tissues. The expression patterns for NtIDDs were distinct. **Figure 7** shows that members with similar expression profiles were clustered together. *NtIDD16/13/11* preferentially expressed in the immature flower. *NtIDD9/21/6/24/3/25/4/22* expressed higher in leaf. *NtIDD10/19* dominantly expressed in capsule. *NtIDD12/2* expressed in all tissues, but still higher in capsule. *NtIDD17/14/8/26* expressed more specifically in root. *NtIDD23* expressed in all tissues, but the expression level in root was the highest. *NtIDD20/18/7* expressed higher in stem. *NtIDD5/15/1* expressed higher in root, stem, and young leaf.

### 3.6 NtIDD response under hormone treatments

**Figure 8:**
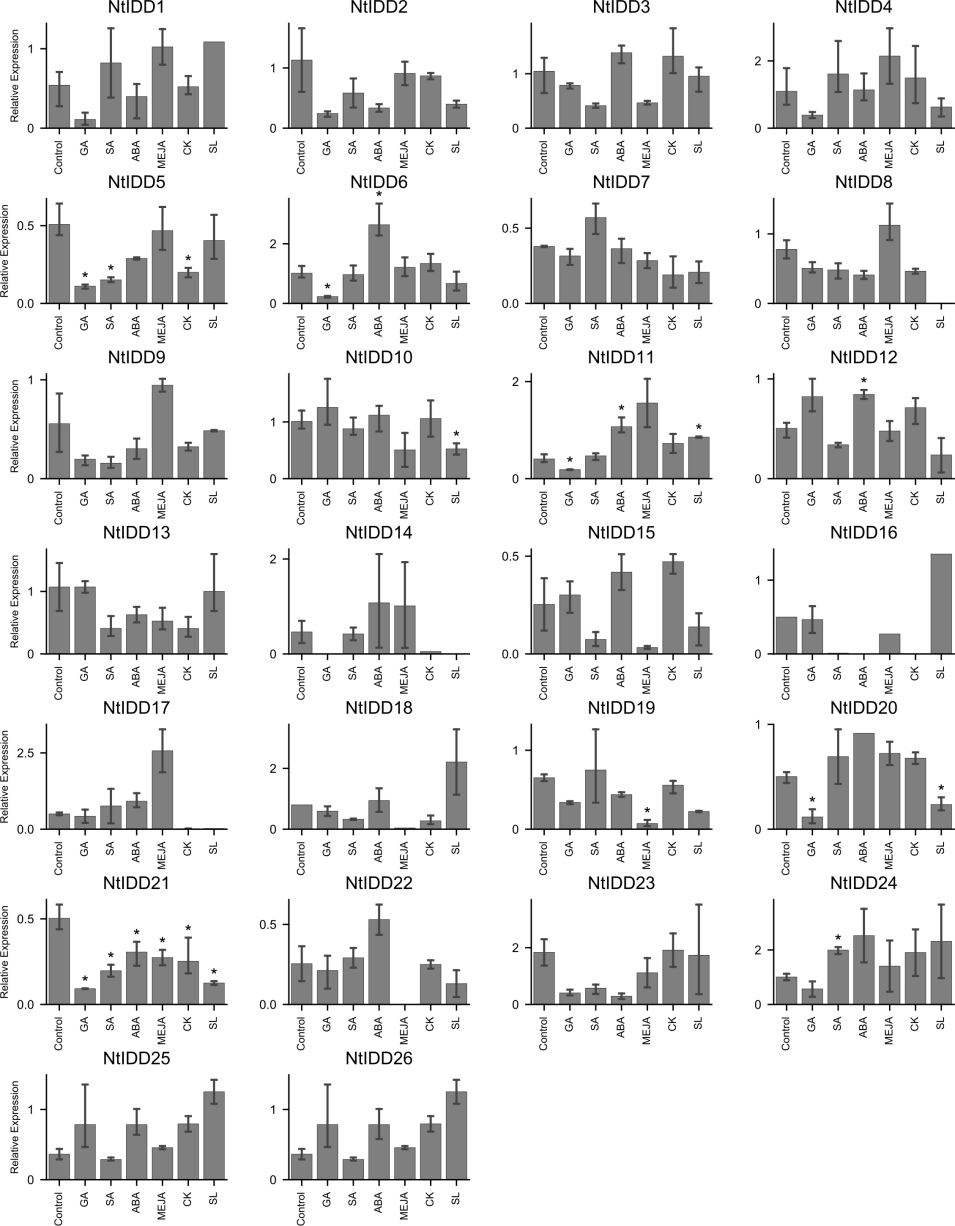
Expression of NtIDDs under exogenous hormone treatments. The error bar represents the mean ± SE. Asterisks show significant differences with *t*-test (*P* < 0.05).

Under conditions with six exogenous hormones, qRT-PCR expression levels of NtIDDs were measured. Compared with the controls, nine NtIDDs showed significant differential expressions under hormone treatments (**Figure 8**). *NtIDD6/11/12/21* differentially expressed under ABA treatment. *NtIDD5/6/11/20/21* were disturbed by GA. *NtIDD5/21* and *NtIDD19/21* responded in CK and MeJA conditions, respectively. *NtIDD5/21/24* were fluctuated by SA application. *NtIDD10/11/20/21* were found mediated under SL treatment.

### 3.7 *NtIDD9* involvement in leaf angle regulation

IDDs were reported functioning in leaf angle regulation. Earlier studies in Arabidopsis characterized the roles of *AtIDD14*, *AtIDD15*, and *AtIDD16* in leaf angle regulation (Cui et al., 2013). Later studies pinpointed *AtIDD4* acting as a direct regulator on *AtIDD14* expression (Völz et al., 2019). Thereby, *AtIDD4* was also implicated in regulating leaf angle. In order to investigate roles of NtIDDs in leaf angle regulation, the ortholog of *AtIDD4* was identified and analyzed.

Based on phylogenetic relationship and sequence similarity, *NtIDD9* was determined as the ortholog of *AtIDD4*. Expression studies in Arabidopsis showed that *AtIDD14* specifically expressed in leaves, *AtIDD15* mainly presented in leaf petioles and stems, and *AtIDD16* and *AtIDD4* highly expressed in leaves and other tissues. In tobacco, as shown above, *NtIDD9* also exhibited high and preferential expression in the leaves. Thus, *NtIDD9* may carry on conserved functions.

**Figure 9:**
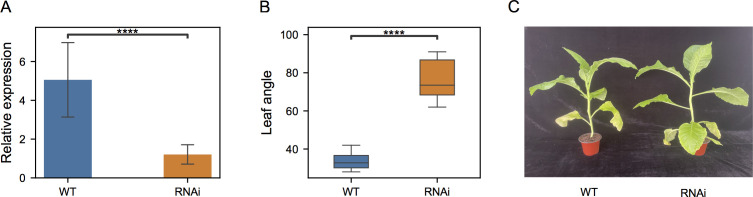
Comparison between wild-type (WT) and *NtIDD9-RNAi* plants. **(A)** qRT-PCR expression levels of *NtIDD9* in wild-type and RNAi plants. The error bar represents the mean ± SE. Asterisks show significant differences with *t*-test (*P* < 1e−4). **(B)** Statistics of leaf angle degrees in wild-type and RNAi plants. The wild-type and transgenic strains in T0 generation were grown in a greenhouse. After 6 weeks, eight plants in each group were selected for measurement. The angles between stem and leaf were determined using a digital angle meter with the unit degrees (°). Asterisks show significant differences with *t*-test (*P* < 1e–4). **(C)** Phenotypes of wild-type and RNAi plants.

To further test its functions in leaf angle, RNA silencing was then performed and *NtIDD9-RNAi* transgenic plants were generated. Quantitative RT-PCR showed that the transcripts of *NtIDD9* were dramatically reduced (**Figure 9A**) in the transgenic RNAi lines, suggesting successful silencing. Further examining the phenotype, it is noticed that the orientation angles of leaves were obviously increased in the RNAi plants (**Figure 9C**). Degree measurement showed that the average leaf angle in the wild type (WT) was 33.8°, whereas it expanded to 76.6° in the RNAi plants (**Figure 9B**). Therefore, the experiment validated *NtIDD9* engaging in leaf angle regulation in tobacco.

### 3.8 Cellular and subcellular localization of *NtIDD9*

**Figure 10:**
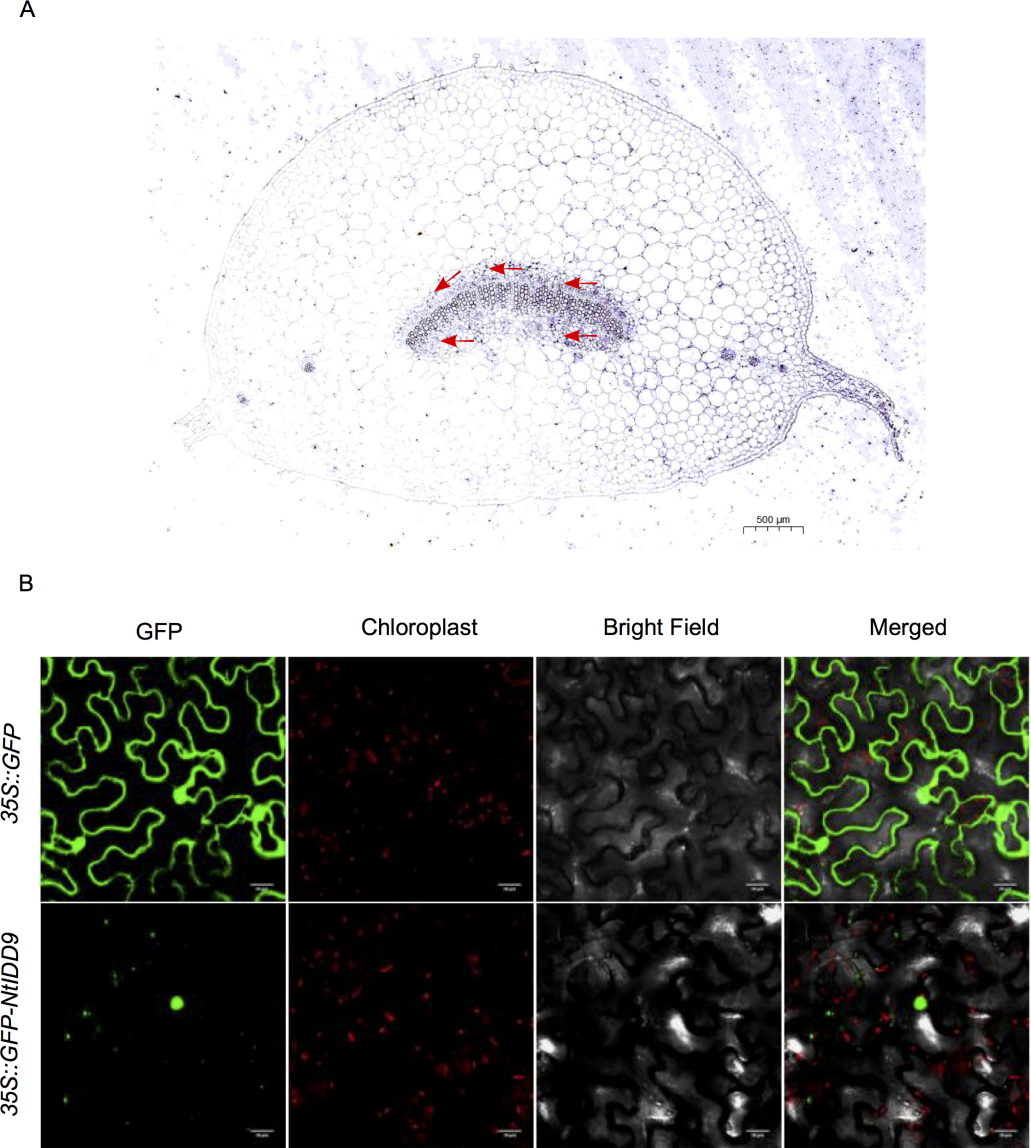
Localization of *NtIDD9*. **(A)** RNA *in situ* hybridization assay of *NtIDD9*. Red arrows indicate endodermis of leaf petiole base. **(B)** Subcellular localization of NtIDD9 protein.

In order to investigate the expression characteristics of *NtIDD9* at the cellular level, RNA *in situ* hybridization technology was used for localization analysis. Results showed that a dense hybridization signal corresponding to *NtIDD9* mRNA concentrated in the endodermis at the base of leaf petiole, which is the site that perceives gravity (Tasaka et al., 1999) (**Figure 10A**).

The subcellular localization of NtIDD9 protein was predicted by using the online tool Cell-PLoc (Chou and Shen, 2010), and it was found located in the nucleus. To determine the actual subcellular localization, NtIDD9 protein fused with green fluorescent protein (GFP) was transiently expressed in tobacco leaves. As shown in **Figure 10B**, the fluorescent signal of 35S::GFP-NtIDD9 fusion protein was restricted to the nucleus, suggesting that the NtIDD9 protein is indeed located in the nucleus.

### 3.9 Plant hormone quantification

**Figure 11:**
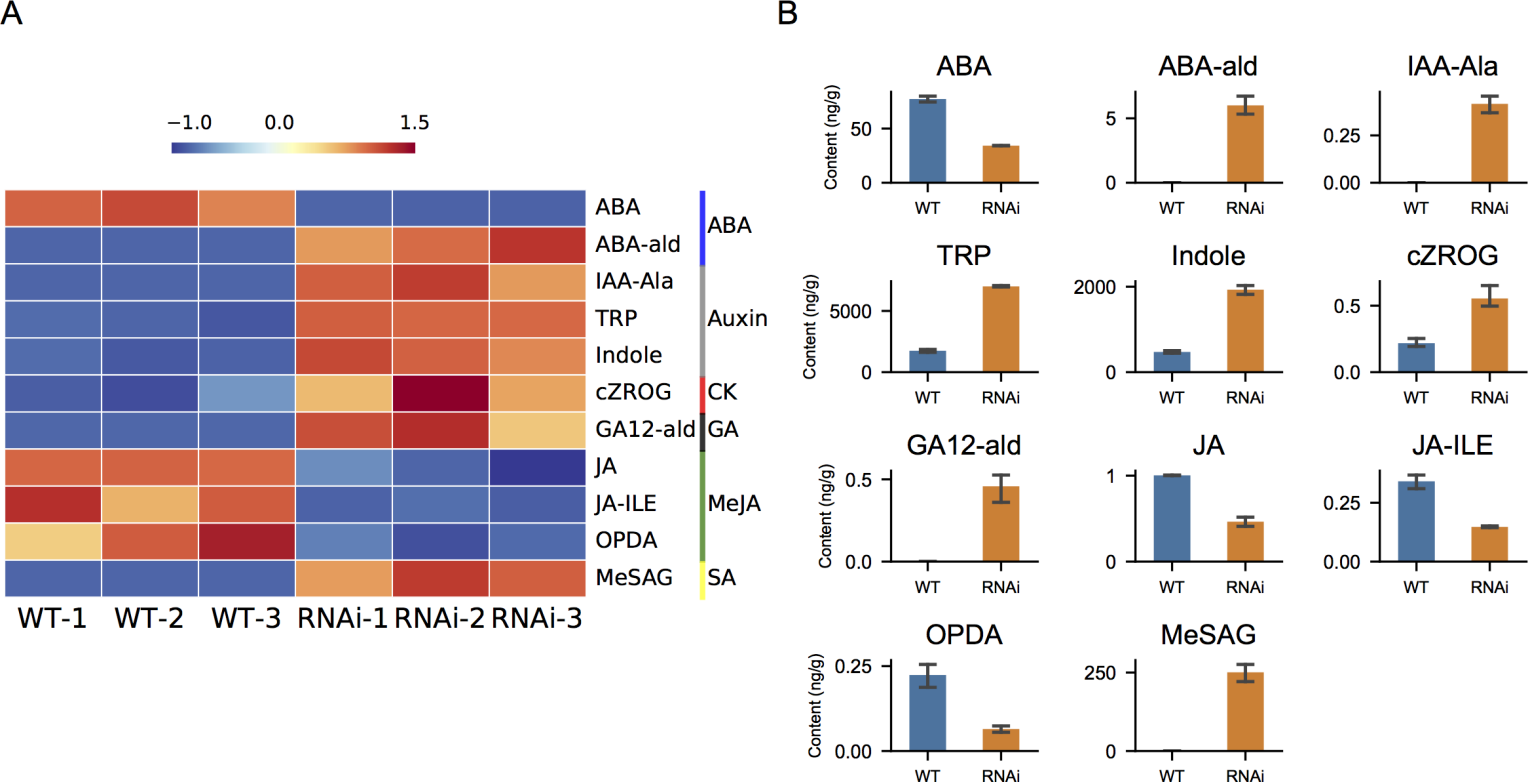
Hormone quantification. **(A)** Heatmap of differential hormone compounds in wild-type and RNAi plants. The color bars on the right denote different hormone classes. **(B)** Levels of differential hormone compounds in wild-type and RNAi plants. The error bar represents the mean ± SE. ABA, abscisic acid; ABA-ald, abscisic aldehyde; IAA-Ala, N-(3-indolylacetyl)-L-alanine; TRP, tryptamine; Indole, indole; cZROG, cis-zeatin-O-glucoside riboside; GA12-ald, gibberellin A12 aldehyde; JA, jasmonic acid; JA-ILE, jasmonoyl-L-isoleucine; OPDA, (cis(+)-12-oxophytodienoic acid; MeSAG, 2-methoxycarbonylphenyl beta-D-glucopyranoside.

Hormone levels were further quantified in wild-type and *NtIDD9-RNAi* plants. Utilizing LC-MS/MS, total 37 compounds belonging to seven hormones were quantified (**Supplementary Table 5**). Further comparison analysis revealed that 11 compounds were significantly different between wild-type and *NtIDD9-RNAi* plants (**Figure 11**). The concentrations of ABA (abscisic acid), JA (jasmonic acid), JA-ILE (jasmonoyl-L-isoleucine), and OPDA (cis(+)-12-oxophytodienoic acid) decreased in RNAi plants. In contrast, the levels of TRP (tryptamine), Indole (indole), and cZROG (cis-zeatin-O-glucoside riboside) increased in RNAi plants. In addition, ABA-ald (abscisic aldehyde), IAA-Ala (N-(3-indolylacetyl)-L-alanine), GA12-ald (gibberellin A12 aldehyde), and MeSAG (2-methoxycarbonylphenyl beta-D-glucopyranoside) were specifically detected in RNAi plants.

## 4 Discussion

In this study, 26 IDDs were identified in tobacco genome. The phylogenetic tree showed that NtIDDs could be classified into five groups. In each group, IDDs from tobacco, Arabidopsis, rice, and maize were clustered. This result indicated that IDDs originated before dicot and monocot speciation. In each group, the numbers of NtIDDs were different with other species. For most cases, there were more NtIDDs in each group than other species, implying that duplications occurred in tobacco.

Gene structure analysis found that most of NtIDDs possessed three to four exons, the intron length varied broadly. Similar results were also found in OsIDDs (Zhang et al., 2020) and ZmIDDs (Feng et al., 2023). Motif scanning showed that motif 1/3 were found in all NtIDDs, motif 2/4/5/6/7 were distributed in most of NtIDDs, while motif 8/9/10 were only found in the subset of NtIDDs. The distribution pattern of motifs revealed conservation and diversification of NtIDD sequences. Motif 1/2/3/8 were associated with the ID domain; thus, these motifs were critical parts for functioning. The functions of other motifs were not clear, however.

Genome location analysis demonstrated that NtIDDs located on 12 chromosomes and 9 scaffolds, revealing scatter distribution of the NtIDD family in the tobacco genome. Based on synteny analysis, 14 NtIDDs were identified involved in segmental and/or WGD duplications, and 12 NtIDDs were classified as dispersed duplications. Tobacco was known as an allopolyploid; thus, the expansion of NtIDDs would be affected by the WGD event. This is common in polyploidy species, such as IDDs in cotton (Ali et al., 2019) and rapeseed (Sun et al., 2022). Dispersed duplications happened with unclear mechanisms but were prevalent in plant genomes (Qiao et al., 2019). Tandem duplications are another type of duplication. It was discovered in cotton IDDs, but not in tobacco, suggesting different duplication modes existed in NtIDDs.

*Cis*-element analysis showed that elements in various stimulus (hormone responsive, stress responsive, and development related) were found in NtIDD promoters. Hormone-responsive elements were associated with abscisic acid, auxin, gibberellin, jasmonate, and salicylic acid; thus, NtIDDs might be coordinated by various hormones. Stress-responsive elements included defense, low-temperature, wounding, drought, and light. This indicated NtIDDs might be affected by external biotic and abiotic stresses. Moreover, development-related elements such as cell cycle and circadian were also detected, implying possible roles of NtIDDs in tobacco growth and development.

RNA-Seq expression analysis showed that NtIDDs expressed widely in tobacco tissues. Most of NtIDDs expressed in multiple tissues. Functional studies on AtIDDs demonstrated distinguished roles in tissues or organs for each member (Prochetto and Reinheimer, 2020). In tobacco, tissue preferential expression patterns were also observed among NtIDDs. *NtIDD16/13/11* might function preferring in young flower. *NtIDD9/21/6/24/3/25/4/22* might play more roles in leaves. *NtIDD10/19/12/2* might participate specific functions in seeds. *NtIDD17/23/14/8/26* might engage in specific roles in roots. *NtIDD20/18/7* might take more functions in stems. *NtIDD5/15/1* might tend to function in roots, stem, and young leaves. These results indicated diversified functions for NtIDDs.

Hormones are important regulators for plant growth and environment stress. Under exogenous hormone applications, several NtIDDs exhibited differential expressions. Among them, *NtIDD10/12/19/24* were found specifically responding in SL, ABA, MeJA, and SA, respectively, suggesting their specific roles in certain hormone signaling, while others, such as *NtIDD5/6/11/20/21*, were disturbed by multiple hormones. For example, *NtIDD20* was affected by both GA and SL. *NtIDD5* was repressed by GA, SA, and CK. Notably, *NtIDD21* was found mediated under all six hormones. Regulating by different hormones indicated their pleiotropic functions in hormone signaling.

*AtIDD14*, *AtIDD15*, and *AtIDD16* were reported to play roles in leaf angle regulation (Cui et al., 2013). *AtIDD4* was also associated due to its direct control on *AtIDD14* expression (Völz et al., 2019). As an ortholog of *AtIDD4*, *NtIDD9* was inferred performing similar functions. In the following experiment, RNA silencing of *NtIDD9* resulted in phenotype of increased leaf angle degree, indicating its involvement in leaf angle regulation in tobacco. Previous studies have shown that the size of the leaf angle is largely determined by gravitropism, which is a gravity-directed growth process (Chen et al., 1999). The process of gravitropism comprises steps such as gravity perception, signal transduction, and growth response (Baldwin et al., 2013). In leaves, endodermis in the basal part of petiole was discovered responsible for gravity perception (Tasaka et al., 1999; Mano et al., 2006). Recent single-cell transcriptome study also found gravitropism-related genes, *LAZY1* (*LA1*), *TILLER ANGLE CONTROL1* (*TAC1*), and *SHOOT GRAVITROPISM6* (*SGR6*) were enriched in the endodermis (Zhang et al., 2021). Analysis in the current study revealed that *NtIDD9* localized in the same cellular part, which suggested its potential role in gravity response. Studies on leaf angle also demonstrated that plant hormones played key roles in regulating angle size (Li et al., 2020; Cao et al., 2022). Endodermis is an important center for hormone signaling. The growth control role for hormones such as auxin, GA, ABA, and SL were gradually illuminating (Dinneny, 2014). In the current study, hormone quantification revealed that concentrations of several classes of hormones were significantly changed in *NtIDD9-RNAi* plants. For instance, auxins (TRP, Indole) were substantially increased in *NtIDD9-RNAi* plants. ABA and JA were significantly reduced in *NtIDD9-RNAi* plants. Meanwhile, GA (GA12-ald) was upregulated in *NtIDD9-RNAi* plants, either. Hence, *NtIDD9* may modulate signal transductions through multiple hormone pathways. Taken together, these findings suggested that *NtIDD9* performed a pivotal role in regulating leaf angle development.

## 5 Conclusion

In summary, a total of 26 IDD genes were identified in tobacco at the genome-scale level. Their phylogenetic relationship, gene structure, sequence motif, genome distribution, duplication mode, and *cis*-elements were systematically analyzed. Tissue expression profiles of NtIDDs showed putative important function in tobacco reproductive and vegetative organs. Exogenous hormone treatment implied their roles under hormone signaling. Functional study revealed *NtIDD9* participated in leaf angle regulation. Taken together, these results lay important foundations for further function and mechanism research for IDDs in tobacco.

## Data Availability

The original contributions presented in the study are included in the article/**Supplementary Material**. Further inquiries can be directed to the corresponding author.

## Supplementary material

Supplementary Table 1 Genes screened by homology and InterPro.

Supplementary Table 2 List of NtIDDs information.

Supplementary Table 3 List of cis-elements in NtIDD promoters.

Supplementary Table 4 Tissue expression values(FPKM) of NtIDDs.

Supplementary Table 5 Hormone levels quantified in wild-type and *NtIDD9-RNAi* plants.

Supplementary Table 6 Primer sequences used for qRT-PCR.
